# Detection of Label-Free Drugs within Brain Tissue Using Orbitrap Secondary Ion Mass Spectrometry as a Complement to Neuro-Oncological Drug Delivery

**DOI:** 10.3390/pharmaceutics14030571

**Published:** 2022-03-05

**Authors:** Phoebe McCrorie, Jonathan Rowlinson, David J. Scurr, Maria Marlow, Ruman Rahman

**Affiliations:** 1 Children’s Brain Tumour Research Centre, Biodiscovery Institute, School of Medicine, University of Nottingham, Nottingham NG7 2RD, UK; mszpm5@exmail.nottingham.ac.uk (P.M.); mszjmr1@exmail.nottingham.ac.uk (J.R.); 2 Advanced Materials and Healthcare Technologies, School of Pharmacy, University of Nottingham, Nottingham NG7 2RD, UK; pazdjs@exmail.nottingham.ac.uk (D.J.S.); maria.marlow@nottingham.ac.uk (M.M.)

**Keywords:** OrbiSIMS, mass spectrometry imaging, glioblastoma, drug delivery

## Abstract

Historically, pre-clinical neuro-oncological drug delivery studies have exhaustively relied upon overall animal survival as an exclusive measure of efficacy. However, with no adopted methodology to both image and quantitate brain parenchyma penetration of label-free drugs, an absence of efficacy typically hampers clinical translational potential, rather than encourage re-formulation of drug compounds using nanocarriers to achieve greater tissue penetration. OrbiSIMS, a next-generation analytical instrument for label-free imaging, combines the high resolving power of an OrbiTrap^TM^ mass spectrometer with the relatively high spatial resolution of secondary ion mass spectrometry. Here, we develop an ex vivo pipeline using OrbiSIMS to accurately detect brain penetration of drug compounds. Secondary ion spectra were acquired for a panel of drugs (etoposide, olaparib, gemcitabine, vorinostat and dasatinib) under preclinical consideration for the treatment of isocitrate dehydrogenase-1 wild-type glioblastoma. Each drug demonstrated diagnostic secondary ions (all present molecular ions [M-H]^−^ which could be discriminated from brain analytes when spiked at >20 µg/mg tissue. Olaparib/dasatinib and olaparib/etoposide dual combinations are shown as exemplars for the capability of OrbiSIMS to discriminate distinct drug ions simultaneously. Furthermore, we demonstrate the imaging capability of OrbiSIMS to simultaneously illustrate label-free drug location and brain chemistry. Our work encourages the neuro-oncology community to consider mass spectrometry imaging modalities to complement in vivo efficacy studies, as an analytical tool to assess brain distribution of systemically administered drugs, or localised brain penetration of drugs released from micro- or nano-scale biomaterials.

## 1. Introduction

Central nervous system (CNS) tumours are a major cause of cancer-related death in children and adults, with high grade invasive brain tumours showing a poor response and frequent high local recurrence rates despite multiple modes of therapy. Conventional oral or intravenous chemotherapy distributes drugs to the whole body, whereby systemic toxicity to healthy parts of the body (e.g., bone marrow failure) limits the maximum dose that can be achieved in the brain. This presents a particular concern for CNS tumours where the blood–brain barrier (BBB) restricts drug influx from the circulation [1,2].

The ability to deliver chemotherapy locally at the tumour site offers the opportunity to target residual cancer cells post-surgery whilst minimising systemic toxicity. We have demonstrated that long-term survival benefits when chemotherapeutics are delivered interstitially post-neurosurgery, via incorporation into polymeric delivery systems [3,4,5]. However, an inherent risk to such drug delivery preclinical studies is the over-reliance on overall survival as an exclusive measure of success. If lack of efficacy is observed predicated upon survival alone, there is uncertainty whether this is due to insufficient brain penetration of drug compounds at therapeutic concentrations, or due to intrinsic resistance of brain tumour cells, despite adequate brain penetration [6].

Historically, the only way to measure the distribution of drugs in the body, including brain, whether delivered locally or systemically, has been to inject radioactive drugs or drug carriers into an animal. The animal is then killed and the location of the drug in the body is worked out by measuring radiation. Unfortunately, this requires a high number of animals, is a method that cannot measure different drugs at the same time, and the radioactive labelling of a drug may mean that the movement of the drug (pharmacokinetics) differs compared to the non-labelled drug that a patient may receive [7,8,9].

In vitro analytical tools which permit both spatial/3D imaging of delivered drugs and semi-quantitation of relative abundance of drug ions can therefore aid a priori screening of candidate drug compounds to ensure those most likely to achieve brain penetration at therapeutic concentrations. Most methods to detect unlabelled drugs within brain tissue use prior homogenisations and extractions such as for high-performance liquid chromatography (HPLC) and liquid chromatograph-mass spectrometry (LC-MS), which prevents acquisition of any spatial information. Increasingly, mass spectroscopy imaging (MSI) techniques have been deployed as an accurate label-free method to detect drugs in the brain at high spatial resolution; for example, matrix-assisted laser desorption ionization (MALDI) has been shown to detect unlabelled small molecule inhibitors (BKM120, RAF265 and Erlotinib) within the brain following systemic administration, and to assess the ability of each drug to cross the blood–brain barrier, as well as their ability to leave the vasculature lumen [10].

More recently, the OrbiSIMS technique, which combines the high resolving power of an OrbiTrap^TM^ (>240,000 at *m*/*z* 200), with the relatively high spatial resolution capability of secondary ion mass spectrometry (SIMS), has emerged as a next-generation analytical instrument for label-free biomedical imaging. OrbiSIMS has been applied to native brain analysis to detect the distribution of neurotransmitters [11], and we have recently demonstrated the ability of OrbiSIMS to identify metabolites which predict relapse in the malignant childhood brain tumour, ependymoma [12].

Here, we develop an ex vivo methodological workflow using OrbiSIMS, to accurately detect brain penetration of drug compounds. We have chosen a panel of drug compounds which exemplify distinct drug chemistries to demonstrate the broad applicability of orbiSIMS. Furthermore, drug compounds were chosen based on feasibility and/or efficacious neuro-oncological application in clinical or pre-clinical settings. Specifically, we have previously demonstrated a long-term survival benefit in high-grade glioma when etoposide is delivered interstitially post-surgery [3]; we have shown the feasibility of olaparib to be incorporated into nanoparticles within a sprayable hydrogel for interstitial brain tumour delivery [13]; vorinostat has recently shown to induce metabolic reprogramming in glioblastoma leading to extended animal survival [14]; gemcitabine significantly enhances glioblastoma survival in animals when delivered interstitially via a lipid nanocapsule-based hydrogel [15]; a recent genome sequencing data has proposed a patient stratification strategy for dasatanib, to potentially enhance therapeutic efficacy in clinical trials [16].

Our work encourages the neuro-oncology community to consider mass spectrometry imaging modalities to complement in vivo efficacy studies, to simultaneously assess brain distribution of systemically administered drugs, or localised brain penetration of drugs, their micro- or nano-scale delivery biomaterial and the relevant native biological tissues in a label-free manner.

## 2. Materials and Methods

### 2.1. Therapeutic Agents

Etoposide (Selleck Chemicals (Houston, TX, USA), Cat. S1225-SEL), olaparib (Selleck Chemicals (Houston, TX, USA), Cat. S1060-SEL) and vorinostat (ApexBio Cat. A4084-APE) were purchased from Stratech, dasatinib from Sigma (Cat CDS023389-25MG), and gemcitabine hydrochloride from Fisher (Cat. 15780899). Drugs were solubilised as follows: dasatinib-30% hydroxypropyl-ß-cyclodextrin (HPßCD); olaparib and etoposide-10% HPßCD; dasatinib and olaparib combined-30% HPßCD; gemcitabine–PBS and vorinostat in DMSO. HPßCD was employed as it aids drug solubility. Cyclodextrins have also been shown to enhance drug distribution such as aiding crocetin over the blood–brain barrier [17]. In our instance, it may also aid drug diffusion in ex vivo brain tissue, which could partially imitate the natural diffusion of drug once delivered locally in vivo; this benefits the development of OrbiSIMS for drug detection in a realistic setting.

### 2.2. Sample Preparation

For optimisation, drug-only controls were generated by solubilising all drugs and spotting onto a non-treated glass slide before drying overnight to remove any moisture. For brain tissue plus drugs, fresh rat brain was homogenised by adding 1 mL distilled water to a whole brain and homogenising using a Stuart SHM1 handheld tissue homogeniser. An amount of 20 mg of this homogenised tissue was spiked with 20 µL, 2 mg/mL drug (final drug concentration 0.5% *w*/*w*), and this was then spotted onto a glass slide alongside control tissue (non-spiked) and dried overnight in a vacuum oven to remove moisture.

For drug penetration analysis, two methods were used: (i) diffusion from tissue immersion; (ii) penetration from a spray device.

We previously determined temperature stability and diffusion timeframe for the drugs of interest using a Franz Cell in vitro diffusion chamber. Stability at only 37 °C was assessed to mimic patient body temperature. Eight hours was sufficient for each drug to diffuse from the Franz Cell donor chamber, through ex vivo mouse brains (1 mm thickness), to the receiver chamber (data not shown). For the immersion method therefore used in this study, a fresh mouse brain was incubated in 3 mL artificial cerebrospinal fluid (CSF) and spiked with different drugs for 8 h at 37 °C. The drugs were solubilised at 2 mg/mL in the carriers listed above. Brains were then placed on dry ice until fully frozen before cryosectioning using a Leica CM3050S cryostat (Leica, Wetzlar, Germany) in 7 µm coronal sections.

For the spray device method, a small pseudo-resection cavity was generated in a fresh ex vivo rat non-disease brain, whereby drug formulations were sprayed into the cavity (50 μL Aptar Pharma device; product code 31068184 and gasket 10276731). The brain was immediately snap-frozen in liquid nitrogen and sectioned into 10 µm sagittal slices using a Leica CM3050S cryostat (Leica, Wetzlar, Germany) at −22 °C. Sections were adhered to gelatin-coated glass microscope slides, before being freeze-dried overnight.

### 2.3. OrbiSIMS Instrumentation

**Figure 1:**
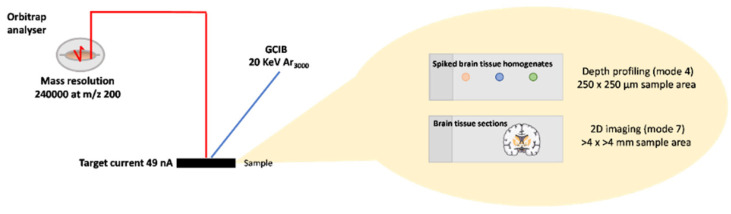
Schematic depicting 3D OrbiSIMS set-up. An ion beam is applied to the surface of a sample, whereby surface molecules are fragmented and ionised. These ions are separated either by time-of-flight or an Orbitrap analyser, following which they are detected to an accuracy of <2 ppm. In our case, the Orbitrap analyser with a mass resolution of 240,000 at *m*/*z* 200 was employed to analyse ions fragmented using a 20 KeV Ar_3000_^+^ ion beam. Samples were analysed using mode 4 and 7 of the instrumentation, as published by Passarelli et al. [11].

**Figure 2:**
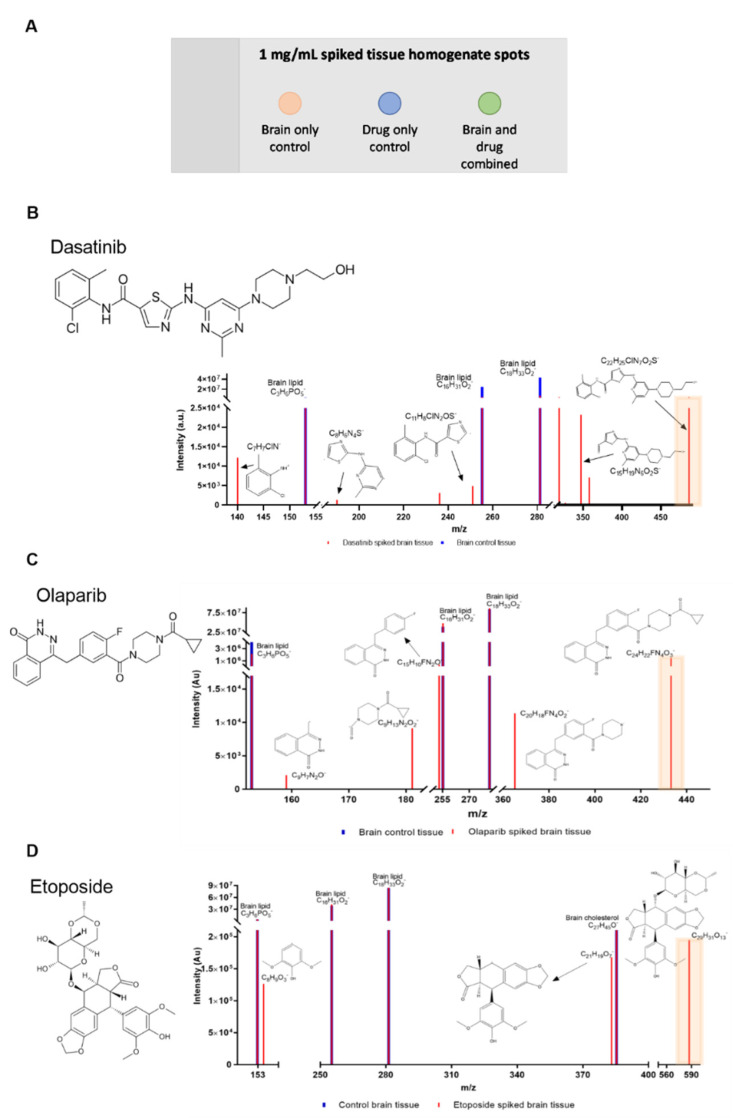
OrbiSIMS spectra for three chemotherapeutics spiked at 1 mg/mL in brain homogenate. (**A**) A schematic to show how the samples were analysed with a brain-only and a drug-only control, (**B**) A spectrum of dasatinib, (**C**) olaparib and (**D**) etoposide. Each spectrum contains the molecular ion peak ([M-H]^−^) highlighted by the tan box (with red peak line), and multiple smaller fragments, differentiating the drugs (red lines) from brain tissue analytes (purple lines). Corresponding exact masses, intensities and standard deviations are shown in supplementary tables.

**Figure 3:**
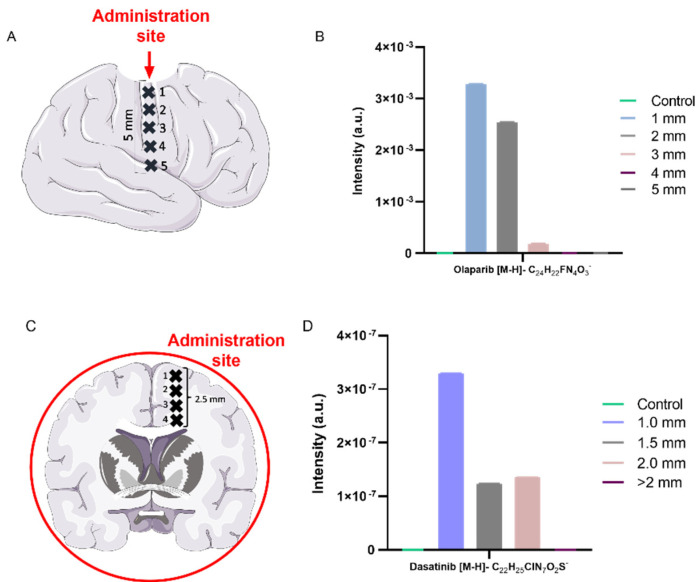
[M-H]^−^ ion intensities for olaparib and dasatinib applied to whole rat or mouse brain ex vivo tissue sections (**A**) Schematic showing the cavity site in a rat brain hemisphere and the corresponding measurement sites at 1 mm increments distal from the administration site and (**B**) olaparib [M-H]^−^ identified at these sites when administered using a spray device. (**C**) Schematic showing the analysis points at ≥0.5 mm increments in a coronal section of a mouse brain bathed in a dasatinib solution. (**D**) Dasatinib [M-H]^−^ identified at these analysis points. Results presented are from a single measurement using 20 KeV Ar_3000_ Orbitrap MS (mode 4).

The OrbiTrap^TM^ Secondary Ion Mass Spectroscopy (OrbiSIMS) technique (schematic shown in Figure 1) utilises a Q Exactive HF for OrbiTrap^TM^ MS (Thermo Fisher, Dreieich, Germany). Mass calibration of the Q Exactive instrument was performed once a day using silver cluster ions. MS spectrum representing analysis of homogenates and single spot analysis upon brain cross-sections (Figure 2 and Figure 3) were obtained in mode 4 of the instrument (shown in ref. [11]) using a 20 keV Ar_3000_^+^ gas cluster ion beam (GCIB) with a target current of 0.3 nA. Briefly, mode 4 utilises single beam Ar_n_ and the Orbitrap analyser to collect a depth profile of the sample. Reference spectra for the control tissue, drug samples and 2 mg/mL drug spiked brain tissue, were accumulated over 250 scans as the ion beam rastered in a sawtooth pattern (250 × 250 µm) in negative mode at the 240,000 at *m*/*z* 200 mass resolution setting. Data was collected between 75 and 1125 Da for each spectrum from spots freeze-dried onto a glass microscope slide.

OrbiSIMS imaging of the brain slices were acquired just below the pseudo-resection cavity site with using the GCIB and OrbiTrap^TM^ (mode 7; ref. [11]) with the same parameters previously specified. Briefly, mode 7 utilises single beam Ar_n_ and the Orbitrap analyser to collect a 2D image of the sample. An ion image containing 208,000 pixels was acquired in macro scan mode over an area of 4000 µm × 4000 µm (pixel size = 20 µm). The total ion image-acquisition time was approximately 6 h per sample. Stage and ion beam rastering (random raster) was used to image the 4.0 mm × 4.0 mm analysis area (pixel size = 20 µm).

The Orbitrap analyser was chosen over the time-of-flight (ToF) analyser due to the enhanced mass resolving power and mass accuracy [11], despite the slower acquisition time.

OrbiSIMS data acquisition and analysis was performed using SurfaceLab 7 (ION-TOF, Germany). Orbitrap MS image analyses were performed using SurfaceLab Version 6.7 (IONTOF, Munster, Germany).

## 3. Results and Discussion

We have tested a panel of brain tumour-relevant drugs within brain tissue to show the scope of OrbiSIMS for label-free detection of drugs. The drugs analysed were vorinostat, dasatinib, gemcitabine, etoposide and olaparib.

Firstly, to determine if drug ions were distinguishable from ions native to brain tissue analytes, drugs were dissolved in a suitable solvent (see methods) and analysed as reference spectra. The drugs were then used to spike rat brain homogenate at 1 mg/mL, alongside brain-only controls for comparison (Figure 2). The optimisation at 1 mg/mL is relevant in local drug delivery as drugs can often be delivered at a higher concentration than when delivered systemically. Our group have delivered 25 mg etoposide locally to rats bearing 9 L glioma with no toxicity observed [3]. Other work has also shown that olaparib delivered at 10 mg locally in rats bearing 9 L glioma was well tolerated (manuscript in preparation).

Mode 4 (depth profiling) was implemented for these analyses to allow for the removal of any surface contamination following sample preparation. This initial surface data is discounted, and the remaining data analysed for peaks. In all cases, the [M-H]^−^ ion was detected at concentrations relevant to delivery and could be resolved from biological peaks. The data showed that OrbiSIMS was much more sensitive in detecting some drugs over others. For example, olaparib exhibited a [M-H]^−^ peak with an area (normalised by total ion intensity) of 1,475,956, whereas etoposide [M-H]^−^ peak had an area of 195,128 (Figure 2C,D). Despite local delivery, potent anti-cancer therapies are still typically administered in low doses due to systemic toxicity, so concentrations are low in the relevant tissue. In ToF-SIMS, the small molecule mass range is greatly dominated by relatively high intensity organic fragment secondary ions which obscure low intensity drug ion peaks. Table S1 shows the characteristic peaks of olaparib and brain lipid fragments (the latter is fully characterised in [11]), with the corresponding peak areas in the olaparib-spiked tissue versus brain-only control. There were seven identifying peaks for olaparib with a mass deviation <2 ppm, and the corresponding peaks can be seen in the OrbiSIMS spectrum in Figure 2C.

Initially the [M-H]^−^ was identified (highlighted with a red background on the spectra) and cross-checked against the brain-only control homogenate to ensure the [M-H]^−^ was diagnostic, before locating likely fragments based on weaker bonds in the structure, i.e., single bonds between aromatic rings. In this case, all five drugs could be distinguished from brain tissue using their molecular ion peak with a mass deviation of below 2 ppm, whilst these peaks were not observed in the brain control tissue. These diagnostic chemical markers were also the most intense ion signals observed for each drug. [M-H]^−^ ions used for the data analysis are ideal, as they represent the full drug structure. Further diagnostic markers were present in each sample from inherent secondary ions formed from the molecular ion. All the peaks identified for the therapeutics were mathematically accurate (i.e., the exact elemental composition could generate the *m*/*z* value of the peak). This was further corroborated by the lack of these peaks being present in the negative control sample. The full detail regarding the peaks observed, including those for gemcitabine and vorinostat, are reported in Figure S1 and Tables S1–S6 in the Supplementary Material. For further work, dasatinib and olaparib were taken forwards due to the molecular ion sensitivity; etoposide was also taken forwards due to previous efficacy shown via local drug delivery [3].

Although previous studies have demonstrated the use of ToF-SIMS [18] and OrbiSIMS [11] to determine brain structure and explore how different sample preparation techniques effect brain tissue by monitoring cholesterol movement [19,20], this study demonstrates the potential for the technique in label-free detection of chemotherapeutics in brain tissue at relevant clinical concentrations. ToF-SIMS has been frequently used to analyse label-free drugs in tissue such as Vitamin C and chlorhexidine in skin [21,22], albeit at much higher concentrations than is relevant in brain drug delivery.

Once the characteristic secondary ion peaks for drug compounds were identified, the peaks could be used to detect drugs throughout whole brain slices. Figure 3 shows how OrbiSIMS can rapidly acquire spectral data at specific points across a brain slice which can be subsequently analysed for characteristic drug ions. The co-ordinates of the spot analysed can be used against the sample holder optical camera image to show the location on the brain slice. In this work, two methodologies were used: the first whereby a pseudo-resection cavity was generated before spraying in a drug solution (olaparib) and snap-freezing to show device-related penetration (Figure 3A,B), and a second whereby a whole brain was bathed in a drug solution (olaparib and/or dasatinib) for 8 h at 37 °C, before snap-freezing to show diffusion of the drug in the brain matter (Figure 3C,D). The detection of drugs from various points across a sample is a relatively rapid means of determining drug presence within the tissue. OrbiSIMS can also generate an ion map image showing the whole distribution of ions across a specific area; however, this takes substantially longer to acquire, and therefore the depth profile at a pre-set position is a practical approach to gain initial drug diffusion data.

As Figure 3 demonstrates, olaparib locally administered by spraying into a pseudo-resection cavity, has shown the [M-H]^−^ ion up to analysis point 4, approximately 4 mm from the cavity wall (administration site). The data also indicates that there is a reduction in ion abundance, proportional to distance away from the resection cavity margin, with no olaparib detectable 5 mm from the administration site. This could be due to no diffusion to this depth, or that the olaparib present is below the limit of detection by OrbiSIMS. We have trialled olaparib spiked in rat brain homogenate down to 25 µg/g and found all peaks are still detectable (Table S3). Morosi et al. have used MALDI-MSI to quantify olaparib and niraparib in ovarian tumour tissue by generating a calibration curve using spots on tissue and deuterated drug as an internal standard, which was comparable to LC-MS/MS acquired data. The study also utilised metal nanoparticles (NPs) to provide a matrix to achieve greater ionisation of the compounds, enabling the limit of detection to be enhanced with TiO_2_NPs (14.7 µg/g (drug/tissue) for niraparib and 22.4 µg/g for olaparib) [23]. Here, we have demonstrated 25 µg/g detection of olaparib in brain tissue without the need of internal standards and additional matrices.

Due to this reduction in peak area when drug ions are sampled distal from the administration site, OrbiSIMS shows the potential to quantify the ion concentrations at each depth analysed. We next assessed whether the peak area could be used against a standard curve (generated by spiking brain tissue homogenates) to show the concentration at the point examined. Although this was trialled in our laboratory with olaparib and dasatinib (Table S1 versus Table S6: 40-fold change in concentration), the peak area observed at each concentration was not relative to the fold-change of the concentrations tested, i.e., a two-fold change in drug concentration did not lead to a two-fold change in peak area. Therefore, to ensure OrbiSIMS can quantify label-free drugs in brain, more concentrations would be needed to complete the data set and generate a curve.

**Figure 4:**
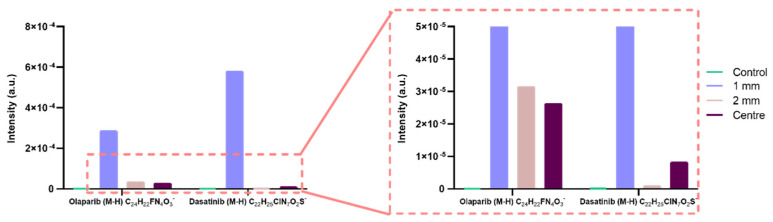
Dual detection of olaparib and dasatinib in a coronal section of a mouse brain. Samples were taken at 1 mm increments distal from the administration site, plus the centre of the coronal section, showing their molecular ions (M-H)^−^ with a deviation of <2 ppm. A mouse brain control section was also analysed for comparison, showing no peak for the molecular ions.

We also utilised the aforementioned bathing method to detect and discriminate two drugs simultaneously in mouse brains bathed in olaparib and dasatinib. After 8 h soaking in drug solution at 37 °C, the brains were snap frozen and sectioned. This data shows successful discrimination between the two drugs in brain tissue (Figure 4) and shows comparable results to Figure 3, whereby the peak area reduces distal from the administration site, again suggesting a reduction in concentration (we found dasatinib presence in samples spiked with 200 µg/mL previously—data not shown).

**Figure 5:**
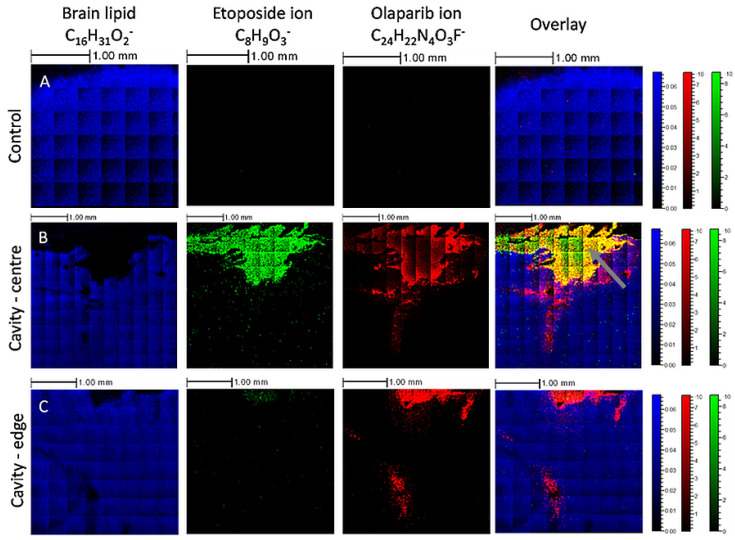
OrbiSIMS secondary ion images showing the location of etoposide and olaparib in ex vivo rat brain tissue following combined spray delivery into a pseudo-resection cavity. Primary ions diagnostic for the olaparib fragment C_24_H_22_N_4_O_3_F^−^ are shown in red, etoposide fragment C_8_H_9_O_3_^−^ shown in green and the brain lipid fragment C_16_H_31_O_2_^−^ shown in blue, plus a combined merged image of the three ions. The secondary ions diagnostic for etoposide and olaparib are also observed to be colocalised within the yellow region (indicated by the grey arrow). Images in (**A**) are from a control slice taken from the edge of the brain distant from the cavity, (**B**) are from the central cavity and (**C**) from the cavity edge.

To establish the capacity to simultaneously image multiple label-free drugs within sectioned brain tissue, etoposide and olaparib were sprayed into a pseudo-resection cavity in ex vivo rat brain before snap-freezing and analysing. A 2D secondary ion image was generated using the GCIB and OrbiTrap^TM^ analyser. An area up to 4 × 4 mm was imaged to generate a chemical ion map across the brain surface, where characteristic ions were located. As seen in Figure 5, olaparib (red) and etoposide (green) were distinguishable from brain matter (blue) and most importantly, ions from each drug could be utilised to investigate permeation into brain tissue following application from a drug delivery device. Although most of the drugs were located in the cavity (due to immediate snap-freezing), there were ions observed up to 2 mm away from the cavity site, in sections both at the centre (Figure 5B) and the edge of the cavity (Figure 5C). The data suggests that olaparib displays a higher permeation into the brain tissue than etoposide, which could be due to a series of factors; one of which is that the LogP of olaparib is higher than that of etoposide (1.9 versus 0.6, respectively), which could aid diffusion into lipophilic areas of the brain [24].

This work demonstrates that the drugs tested can be imaged at a resolution compatible with distinguishing them in different areas of a brain section. To enhance this work further, recently demonstrated ToF-SIMS capability of differentiating glioblastoma tumour tissue from brain tissue [25], could also be extrapolated to OrbiSIMS, whereby drugs could be analysed within brain tumour regions to assess drug penetration into brain parenchyma harbouring tumour. This information would further enhance drug discovery efforts via gaining an understanding in drug penetration and efficacy.

## 4. Conclusions

This data provides insight to the capabilities of OrbiSIMS technique in the application of detecting drugs, including etoposide, dasatinib and olaparib simultaneously within rat brain tissue. This work demonstrates a novel approach to the analysis of label-free ex vivo tissue discrimination of drug compounds from brain tissue analytes using OrbiSIMS and opens an avenue to complement in vivo systemic and localised brain tumour drug delivery studies, specifically the semi-quantitative measurement of drug diffusion distance through brain parenchyma.

Our findings potentially help address the unmet scientific and clinical challenge within neuro-oncology research, of determining whether therapeutic drug concentrations have penetrated brain regions harbouring residual tumour, and which are responsible for recurrence manifestation. For example, tissue at various distances from a surgical resection margin could be serially removed using a biopsy punch and tissue homogenised, followed by OrbiSIMS analyses as described here. The methodology could also be applied to post-sacrificial in vivo tissue cryosections (non-homogenised) and OrbiSIMS imaging conducted to gain complementary spatial information regarding diffused drugs.

## Data Availability

Data is contained within the article or Supplementary Material.

## Supplementary Materials

The following supporting information can be downloaded at: www.mdpi.com/article/10.3390/pharmaceutics1596046/s1. Figure S1: OrbiSIMS spectra for vorinostat and gemcitabine at 1 mg/mL in brain homogenate; Table S1: Characteristic orbiSIMS peaks of 20 mL olaparib at 1 mg/mL with 20 mg homogenised rat brain tissue (final drug concentration = 0.5% [*w*/*w*]); Table S2: Characteristic orbiSIMS peaks of 20 mL etoposide at 1 mg/mL with 20 mg homogenised rat brain tissue (final drug concentration = 0.5% [*w*/*w*]); Table S3: Characteristic orbiSIMS peaks of 20 mg/mL olaparib in homogenised rat brain tissue; Table S4: Characteristic orbiSIMS peaks of 20 mL vorinostat at 1 mg/mL with 20 mg homogenised rat brain tissue (final drug concentration = 0.5% [*w*/*w*]); Table S5: Characteristic orbiSIMS peaks of 20 mL gemcitabine at 1 mg/mL with 20 mg homogenised rat brain tissue (final drug concentration = 0.5% [*w*/*w*]); Table S6: Characteristic orbiSIMS peaks of 20 mL dasatinib at 1 mg/mL with 20 mg homogenised rat brain tissue (final drug concentration = 0.5% [*w*/*w*]).
