# Supplementary material for: Detection of Label-Free Drugs within Brain Tissue Using Orbitrap Secondary Ion Mass Spectrometry as a Complement to Neuro-Oncological Drug Delivery
Source: Pharmaceutics. 2022 Mar 5;14(3):571. doi: 10.3390/pharmaceutics14030571 (PMC8953756; doi:10.3390/pharmaceutics14030571)
Supplement: Supplementary file 1 [file pharmaceutics-14-00571-s001.zip › pharmaceutics-1596046-supplementary.pdf]

pharmaceutics

MDPI

Supplementary Materials: Detection of Label-Free Drugs within Brain Tissue Using Orbitrap Secondary Ion Mass Spectrometry as a Complement to Neuro-Oncological Drug Delivery

Phoebe McCrorie, Jonathan Rowlinson, David J. Scurr, Maria Marlow and Ruman Rahman

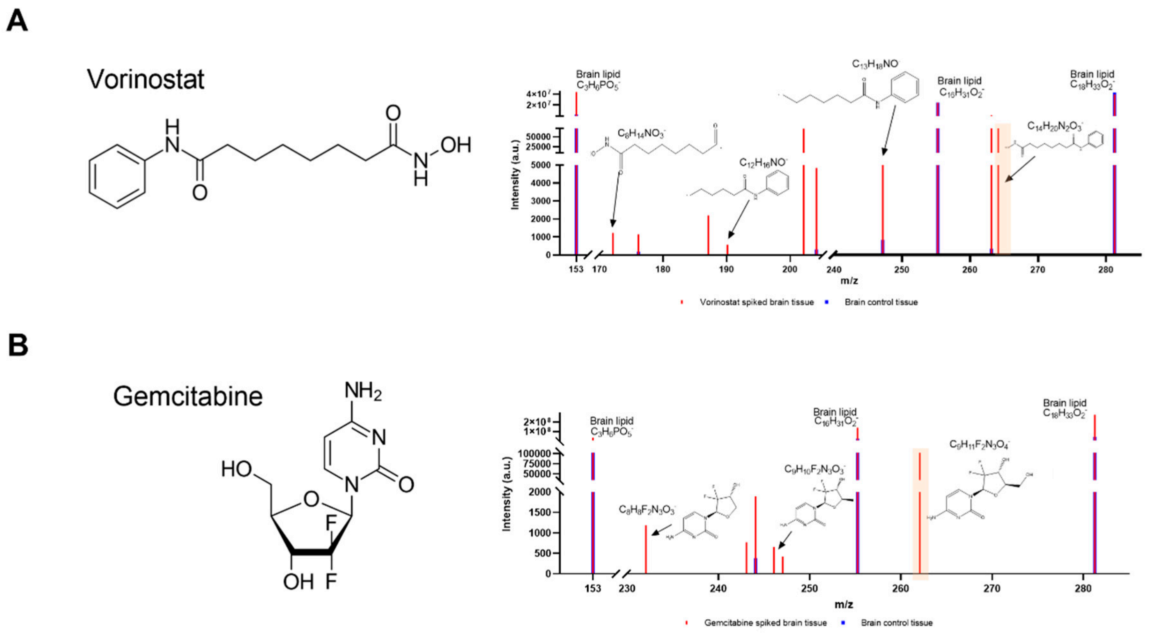

Figure S1. OrbiSIMS spectra for (**A**) vorinostat and (**B**) gemcitabine at 1mg/mL in brain homogenate. Both spectra contain the molecular ion peak (M-H) highlighted by the red highlighted box, and multiple smaller fragments, differentiating the drugs from brain tissue analytes. Corresponding exact masses, intensities and standard deviations are shown in supplementary tables. Results presented are from a single measurement using 20 KeV Ar3000 Orbitrap MS (mode 4).

Table S1. Characteristic orbiSIMS peaks of 20 mL olaparib at 1 mg/ml with 20 mg homogenised rat brain tissue (final drug concentration= 0.5% [w/w]).

				Brain control		Olaparib + Brain	
**No.**	**Center Mass (u)**	**Assignment**	**Description**	**Area**	**Mass deviation (ppm)**	**Area**	**Mass deviation (ppm)**
**1**	253.0783	$C_{15}H_{10}FN_2O^-$	Fragment	0	+12.3	344242	+0.4
**2**	433.1677	$C_{24}H_{22}FN_4O_3^-$	Molecular ion	0	-8.2	1475956	-1.1
**3**	145.0406	$C_8H_5N_2O^-$	Fragment	93	-4.2	14100	-1.2
**4**	159.0561	$C_9H_7N_2O^-$	Fragment	0	+5.3	2111	-1.5
**5**	181.0982	$C_9H_{13}N_2O_2^-$	Fragment	0	-4.2	9125	-0.5
**6**	153.1032	$C_8H_{13}N_2O^-$	Fragment	0	-4.6	7751	-1.1
**7**	365.1415	$C_{20}H_{18}FN_4O_2^-$	Fragment	0	+1.3	11365	-1.1
**8**	255.2329	$C_{16}H_{31}O_2^-$	Brain lipid	38992508	+1.7	47806478	+0.4
**9**	152.9957	$C_3H_6PO_5^-$	Brain lipid	4075584	+0.3	2167331	-1.1
**10**	281.2486	$C_{18}H_{33}O_2^-$	Brain lipid	84148602	+1.3	86463483	-0.0
**11**	385.3473	$C_{27}H_{45}O^-$	Brain cholesterol	542562	+0.4	560758	-0.8

Table S2. Characteristic orbiSIMS peaks of 20 mL etoposide at 1 mg/ml with 20 mg homogenised rat brain tissue (final drug concentration = 0.5% [w/w]).

				Brain control		Etoposide + Brain	
**No.**	**Center Mass (u)**	**Assignment**	**Description**	**Area**	**Mass Deviation (ppm)**	**Area**	**Mass Deviation (ppm)**
**1**	587.176476	$C_{29}H_{31}O_{13}^-$	Molecular ion	0	+10.1	195128	-0.0
**2**	435.129253	$C_{21}H_{23}O_{10}^-$	Fragment	0	+4.5	257	+0.3
**3**	153.055473	$C_8H_9O_3^-$	Fragment	0	-2.0	126371	-1.6
**4**	383.113165	$C_{21}H_{19}O_7^-$	Fragment	0	-5.7	168101	+0.2
**5**	255.2329	$C_{16}H_{31}O_2^-$	Brain lipid	38992508	+1.7	42640092	+1.4
**6**	152.9957	$C_3H_6PO_5^-$	Brain lipid	4075584	+0.3	4080847	-0.0
**7**	281.2486	$C_{18}H_{33}O_2^-$	Brain lipid	84148602	+1.3	84980993	+1.1
**8**	385.3473	$C_{27}H_{45}O^-$	Brain cholesterol	542562	+0.4	420006	+0.1

Table S3. Characteristic orbiSIMS peaks of 20 mg/mL olaparib in homogenised rat brain tissue

No.	Center Mass (u)	Assignment	Description	Brain control		Olaparib + Brain	
				Area	Mass deviation (ppm)	Area	Mass deviation (ppm)
1	253.0783	$C_{15}H_{10}FN_2O^-$	Fragment	0	+12.3	8130	
2	433.1677	$C_{24}H_{22}FN_4O_3^-$	Molecular ion	0	-8.2	68148	
3	145.0406	$C_8H_5N_2O^-$	Fragment	93	-4.2	91220	
4	159.0561	$C_9H_7N_2O^-$	Fragment	0	+5.3	35794	
5	181.0982	$C_9H_{13}N_2O_2^-$	Fragment	0	-4.2	259033	
6	153.1032	$C_8H_{13}N_2O^-$	Fragment	0	-4.6	154525	
7	365.1415	$C_{20}H_{18}FN_4O_2^-$	Fragment	0	+1.3	95	
8	255.2329	$C_{16}H_{31}O_2^-$	Brain lipid	38992508	+1.7	34119796	
9	281.2486	$C_{18}H_{33}O_2^-$	Brain lipid	84148602	+1.3	52791661	
10	385.3473	$C_{27}H_{45}O^-$	Brain cholesterol	542562	+0.4	4664140	

Table S4. Characteristic orbiSIMS peaks of 20 mL vorinostat at 1 mg/ml with 20 mg homogenised rat brain tissue (final drug concentration = 0.5% [w/w]).

No.	Center Mass (u)	Assignment	Description	Brain control		Vorinostat + brain	
				Area	Mass deviation (ppm)	Area	Mass deviation (ppm)
1	263.1397	$C_{14}H_{19}N_2O_3^-$	M-H	353	-1.5	1687335	-0.6
2	264.1431	$C_{14}H_{20}N_2O_3^-$	M	0	-16.4	166727	-1.4
3	202.1244	$C_{13}H_{16}NO^-$	Fragment	311	3.1	70003	-1.1
4	176.1083	$C_{11}H_{14}NO^-$	Fragment	176	1.4	1145	-1.9
5	190.1228	$C_{12}H_{16}NO^-$	Fragment	0	-4.7	563	-0.9
6	204.1394	$C_{13}H_{18}NO^-$	Fragment	306	0.1	4835	-1.1
7	247.1452	$C_{14}H_{19}N_2O_2^-$	Fragment	848	-0.1	10259	-0.8
8	172.0978	$C_8H_{14}NO_3^-$	Fragment	494	2.7	1229	1.2
9	187.1096	$C_8H_{15}N_2O_3^-$	Fragment	0	4.1	2209	-1.1
10	255.2339	$C_{16}H_{31}O_2^-$	Brain lipid	24792697	1.2	24431231	0.0
11	281.2486	$C_{18}H_{33}O_2^-$	Brain lipid	42916135	0.9	38923936	-0.4
12	385.3473	$C_{27}H_{45}O^-$	Brain cholesterol	3582325	-0.3	43333052	-1.5

Table S5. Characteristic orbiSIMS peaks of 20 mL gemcitabine at 1 mg/ml with 20 mg homogenised rat brain tissue (final drug concentration = 0.5% [w/w]).

No.	Center Mass (u)	Assignment	Description	Brain control		Gemcitabine + brain	
				Area	Mass deviation (ppm)	Area	Mass deviation (ppm)
**1**	262.0650	$C_9H_{10}F_2N_3O_4^-$	Molecular ion	0	2	101904	1.2
**2**	247.0539	$C_9H_9F_2N_2O_4^-$	Fragment	0	3.5	419	0.4
**3**	246.0704	$C_9H_{10}F_2N_3O_3^-$	Fragment	0	3.4	658	-0.3
**4**	232.0544	$C_8H_8F_2N_3O_3^-$	Fragment	0	1.9	1189	-1.6
**5**	244.0731	$C_9H_{11}FN_3O_4^-$	Fragment	387	-3.2	1892	-0.3
**6**	243.0657	$C_9H_{10}FN_3O_4^-$	Fragment	0	0.7	774	0.0
**9**	255.2339	$C_{16}H_{31}O_2^-$	Brain lipid	24792697	1.2	140961907	2.1
**10**	281.2486	$C_{18}H_{33}O_2^-$	Brain lipid	42916135	0.9	277414449	1.8
**11**	385.3473	$C_{27}H_{45}O^-$	Brain cholesterol	3582325	-0.3	35431934	0.5

Table S6. Characteristic orbiSIMS peaks of 20 mL dasatinib at 1 mg/ml with 20 mg homogenised rat brain tissue (final drug concentration = 0.5% [w/w]).

No.	Center Mass (u)	Assignment	Description	Brain control		Dasatinib + brain	
				Area	Mass deviation (ppm)	Area	Mass deviation (ppm)
**1**	261.1457	$C_{12}H_{17}N_6O^-$	Fragment	0	-4.8	7466588	0.6
**2**	231.1375	$C_{11}H_{15}N_6^-$	Fragment	396	7.1	690695	1.8
**3**	217.1194	$C_{10}H_{13}N_6^-$	Fragment	0	-6.0	938722	-0.8
**4**	174.0786	$C_8H_8N_5^-$	Fragment	1151	3.6	1676612	1.0
**5**	172.0628	$C_8H_6N_5^-$	Fragment	3692	2.9	1131926	-0.6
**6**	160.0627	$C_7H_6N_5^-$	Fragment	17561	2.7	7009838	-0.5
**7**	319.1326	$C_{14}H_{19}N_6OS^-$	Fragment	0	-4.6	4466339	1.1
**8**	486.1449	$C_{22}H_{25}ClN_7O_2S^-$	M-H	0	-6.2	4444771	1.0
**9**	255.2339	$C_{16}H_{31}O_2^-$	Brain lipid	24792697	1.2	4787061	0.5
**10**	281.2486	$C_{18}H_{33}O_2^-$	Brain lipid	42916135	0.9	7523922	0.3
**11**	385.3473	$C_{27}H_{45}O^-$	Brain cholesterol	3582325	-0.3	2268736	-0.8
